# Ploidy and Implantation Potential: Non-Invasive Small Non-Coding RNA-Based Health Assessment of Day 5 and 6 Blastocysts

**DOI:** 10.3390/ijms262412102

**Published:** 2025-12-16

**Authors:** Angelika V. Timofeeva, Ivan S. Fedorov, Guzel V. Savostina, Alla M. Tarasova, Svetlana G. Perminova, Tatyana A. Nazarenko, Gennady T. Sukhikh

**Affiliations:** National Medical Research Center for Obstetrics, Gynecology and Perinatology Named After Academician Kulakov V.I., 117997 Moscow, Russia

**Keywords:** blastocyst, euploidy, aneuploidy, implantation potential, piwiRNA, culture medium, real-time PCR, logistic regression model, assisted reproductive technologies

## Abstract

A predominant etiological factor in implantation failure and early pregnancy loss is embryonic chromosomal abnormalities. The current clinical standard for determining embryonic ploidy is invasive preimplantation genetic testing. This procedure imposes mechanical stress on embryonic cells during trophectoderm biopsy and fails to significantly improve live birth rates per transfer, likely due to its inability to evaluate the embryo’s implantation potential. Consequently, there is a clear need to develop a non-invasive method, suitable for routine clinical practice, that can simultaneously assess both the ploidy and implantation competence of a blastocyst prior to uterine transfer. Our research group was the first to achieve this by quantifying specific piwiRNAs (piR_016677, piR_017716, piR_020497, piR_015462) in spent culture medium. These data served as the foundation for logistic regression models tailored for day 5 blastocysts, day 6 blastocysts, and blastocysts irrespective of their developmental rate. These models demonstrated high diagnostic accuracy, with specificity ranging from 68% to 100% and sensitivity from 71% to 100%. The rationale for employing these molecules as biomarkers lies in their potential biological roles, which encompass maintaining genomic stability through *LINE-1* regulation, as well as direct involvement in critical processes such as cell cycle control, spindle assembly, and cellular adhesion—all of which are imperative for successful implantation.

## 1. Introduction

Infertility represents a global health challenge, affecting an estimated 10–15% of couples of reproductive age worldwide [1]. While in vitro fertilization (IVF) programs have achieved considerable advancements in addressing infertility—through optimized embryo culture conditions [2,3], refined morphological and molecular criteria for quality assessment [4], and improved selection of the optimal developmental stage for uterine transfer [5,6]—pregnancy rates remain suboptimal. According to data from the Russian Association of Human Reproduction, success rates in IVF cycles do not exceed 30–49% [7]. It is well-established that over half of early pregnancy losses are associated with aneuploid embryos [8]. Moreover, the incidence of embryos with chromosomal aberrations exhibits a strong correlation with maternal age. The proportion of aneuploid embryos rises from 20 to 27% in women aged 26–30 years to as high as 95.5% in women aged 45 years [9,10]. Considering the contemporary trend toward delayed childbearing, the preimplantation identification of chromosomally abnormal embryos is of paramount importance.

The current clinical standard for assessing embryonic ploidy is invasive preimplantation genetic testing for aneuploidy (iPGT-A) [11]. This technique necessitates trophectoderm biopsy for chromosomal copy number variation analysis, an invasive procedure that induces mechanical stress on embryonic cells. This stress manifests as DNA budding and shedding from nuclei, significantly exceeding the levels observed during physiological blastocoel expansion and hatching [12]. Consequently, the biopsy procedure itself may contribute to the occurrence of mosaic aneuploidy, in addition to errors in mitotic chromosome segregation. Furthermore, a multicenter randomized clinical trial [13] demonstrated no significant improvement in live birth rates per transfer among women aged 25–40 years following the transfer of a euploid embryo selected via iPGT-A, compared to selection based solely on morphological criteria (50% vs. 46%; *p* = 0.32). This finding implies that the blastocyst’s capacity for endometrial apposition, adhesion, and invasion is governed not only by cellular ploidy but also by a multitude of critical molecular mechanisms originating from both the blastocyst and the maternal endometrium, which collectively orchestrate a successful embryo–endometrial dialog [14,15].

A principal limitation of iPGT-A is its inability to reliably distinguish between uniformly aneuploid and mosaic embryos. Evidence suggests that a subset of embryos diagnosed as aneuploid or mosaic by iPGT-A can result in the birth of healthy, euploid infants [16,17,18], likely because the biopsy of 5–6 trophectoderm cells may not be representative of the entire embryo’s chromosomal constitution. This underscores the necessity for alternative, preferably non-invasive, methods of embryo quality assessment. A comprehensive review by Silvia Toporcerová systematically analyzed the potential of various components of the embryonic secretome as a non-invasive platform for preimplantation testing and IVF outcome prediction [19]. These components include extracellular genomic DNA, mitochondrial DNA, mRNA, long non-coding RNAs, small RNAs, proteins (e.g., VEGF-A, IL-6, histidine-rich glycoprotein HRG, EMMPRIN, HLA-G, various interleukins, LIF, GM-CSF, JARID2, hCG isoforms), and amino acids (e.g., leucine, alanine, serine). While the analysis of extracellular genomic DNA (ni-PGT-A)—released into the spent culture medium via apoptosis and within extracellular vesicles—demonstrates fewer diagnostic errors regarding embryonic mosaicism compared to iPGT-A (with 78% concordance between the methods), ni-PGT-A similarly fails to inform on the blastocyst’s implantation potential. For other secretome components, the review concludes that a lack of reproducibility, validation, and robust clinical evaluation currently precludes their practical application, highlighting the need for further large-scale randomized controlled trials.

A promising frontier in reproductive biology involves the analysis of piwi-interacting RNAs (piwiRNAs), a class of small non-coding RNAs, in the spent embryo culture medium. piwiRNAs serve as crucial “guardians of the genome” by mediating transcriptional and post-transcriptional silencing of mobile genetic elements [20,21]. Consequently, an imbalance in piwiRNA composition could lead to genomic instability through the increased activity of repetitive sequences. Furthermore, PIWI proteins are instrumental in regulating the proliferation and maintenance of germline stem cells, the progenitors of oocytes and spermatozoa [22,23]; the quality of these gametes is a primary determinant of subsequent embryonic developmental potential. Our research group was the first to identify a correlation between the levels of specific piwiRNAs in the spent culture medium and the quality of morula and blastocyst stage embryos across various morphological grades, paving the way for a non-invasive test system to determine implantation potential independent of karyotype [24,25,26].

In a recent investigation utilizing deep sequencing of small non-coding RNAs followed by quantitative RT-PCR validation, we analyzed spent media from day 5 blastocysts with known iPGT-A results and subsequent ART outcomes after euploid blastocyst transfer. This work yielded logistic regression models capable of identifying euploid blastocysts with high implantation potential based on the levels of specific extracellular piwiRNAs [27]. The objective of the present study was to validate these identified marker piwiRNAs using an independent cohort of samples derived from spent culture media of day 5 blastocysts and from that of developmentally delayed embryos that formed late blastocysts on day 6 post fertilization.

## 2. Results

To validate the marker extracellular piwiRNAs previously identified [27]—which differentiate high-implantation-potential euploid day 5 blastocysts (EuBl, impl) from lower-quality counterparts, namely euploid blastocysts that failed to implant (EuBl, non-impl) and aneuploid blastocysts (AneuBl)—an independent sample set was utilized. This set comprised 183 spent culture medium samples obtained from 110 couples. All included embryos underwent PGT-A with parallel collection of spent culture medium, performed either on day 5 post-fertilization (for embryos reaching the late blastocyst stage) or on day 6 post-fertilization (for embryos demonstrating developmental delay, presenting as morulae or early blastocysts on day 5; see Table S1). Given our prior findings that developmental delay influences the profile of extracellular small non-coding RNAs [25], the samples were stratified into two primary groups: one group (*n* = 141) consisted of late blastocysts formed on day 5 (20 EuBl, impl; 40 EuBl, non-impl; 83 AneuBl), and the other (*n* = 42) consisted of late blastocysts formed on day 6 (8 EuBl, impl; 6 EuBl, non-impl; 21 AneuBl). The piwiRNAs piR_016677, piR_017716, piR_020497, and piR_015462 were selected as marker molecules for assessing ploidy and implantation potential across all 183 samples, with piR_022258 serving as the endogenous reference RNA, consistent with our previous study [27].

### 2.1. Analysis of Spent Culture Media from Day 5 Blastocysts by Quantitative RT-PCR

**Figure 1:**
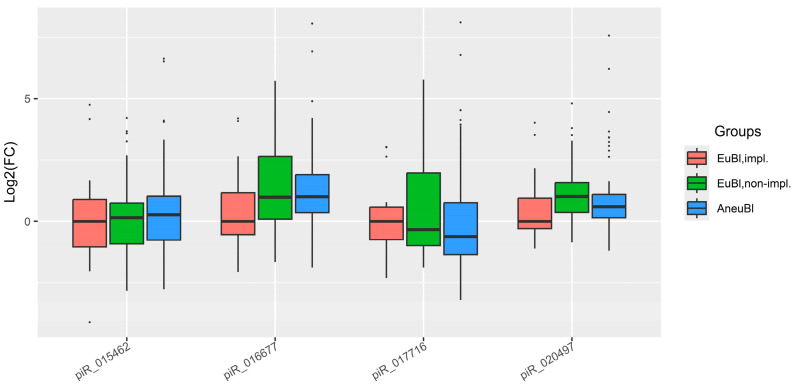
Box plot of the log2(fold change) in piwiRNA levels in spent culture media from day 5 blastocysts relative to the median “∆∆Ct” value in the “EuBl, impl” group. “EuBl, impl”: euploid blastocysts with implantation potential; “EuBl, non-impl”: euploid blastocysts without implantation potential; “AneuBl”: aneuploid blastocysts.

To quantify changes in the levels of piR_016677, piR_017716, piR_020497, and piR_015462 in spent media from day 5 blastocysts, the fold change in the “AneuBl” and “EuBl, non-impl” groups relative to the “EuBl, impl” group was calculated. The “∆Ct” value for each marker piwiRNA was determined using piR_022258 for normalization. These values were then subtracted from the corresponding “∆Ct” values obtained from the control culture medium (from the same lot and incubated under identical conditions), yielding the “∆∆Ct” values. The median “∆∆Ct” value from the “EuBl, impl” group was subsequently subtracted from the “∆∆Ct” value of each individual sample across all groups, resulting in the log2(fold change) for each piwiRNA. These values are presented in the box plot in Figure 1. The analysis revealed that the spent media from poor-quality day 5 blastocysts (EuBl, non-impl and AneuBl) were characterized by elevated levels of piR_015462, piR_016677, and piR_020497, and a reduced level of piR_017716 compared to the high-quality (EuBl, impl) group. Statistically significant differences (*p* < 0.05) were observed for piR_016677 and piR_020497 (Table 1).

**Figure 2:**
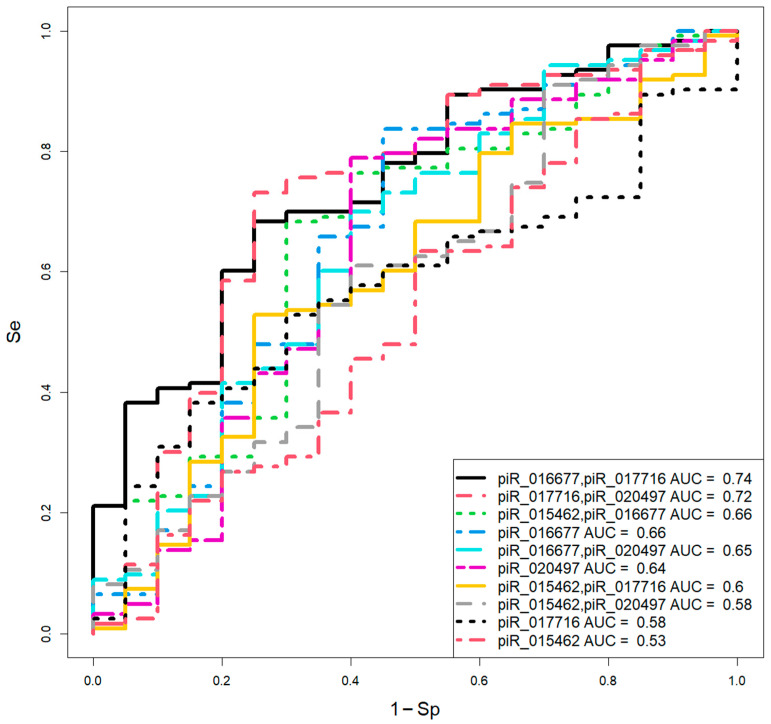
Logistic regression models for assessing day 5 blastocyst quality based on the log2 (fold change) of piwiRNA levels in spent culture media relative to the median “∆∆Ct” in the “EuBl, impl” group.

To evaluate the diagnostic significance of these piwiRNAs for day 5 blastocyst quality, logistic regression models were constructed using the log2(fold change) values relative to the “EuBl, impl” median. Stepwise feature selection in RStudio 2025.09.2 + 418 “Cucumberleaf Sunflower” identified optimal piwiRNA combinations, with blastocyst quality (0 = EuBl, impl; 1 = EuBl, non-impl or AneuBl) as the dependent variable (Figure 2).

The model parameters (Table 2) indicated that the combinations of piR_016677 and piR_017716 (Se = 69%, Sp = 75%) and piR_017716 and piR_020497 (Se = 73%, Sp = 75%) yielded the best diagnostic accuracy.

### 2.2. Analysis of Spent Culture Media from Day 6 Blastocysts by Quantitative RT-PCR

**Figure 3:**
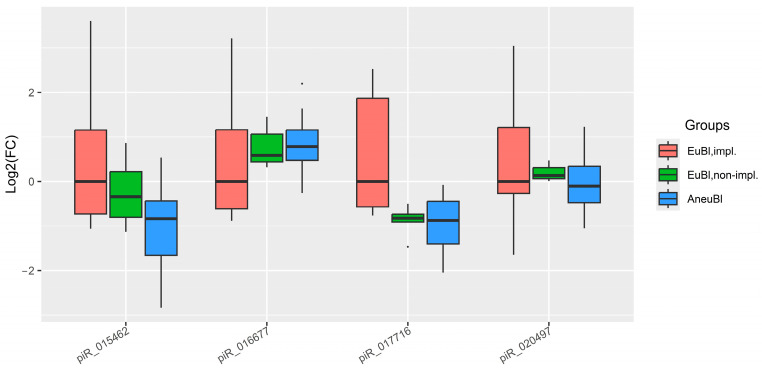
Box plot of the log2(fold change) in piwiRNA levels in spent culture media from day 6 blastocysts relative to the median “∆∆Ct” value in the “EuBl, impl” group. Group definitions are as in Figure 1.

The fold change in piwiRNA levels (piR_016677, piR_017716, piR_020497, piR_015462) for day 6 blastocysts was calculated as described in Section 2.1. The results are displayed in the box plot in Figure 3. The spent media from poor-quality day 6 blastocysts (EuBl, non-impl and AneuBl) exhibited decreased levels of piR_015462 and piR_017716, and increased level of piR_016677 compared to the high-quality (EuBl, impl) group. Statistically significant differences (*p* < 0.05) were observed for piR_017716 and piR_015462, and no significant change—for piR_020497 (Table 3).

**Figure 4:**
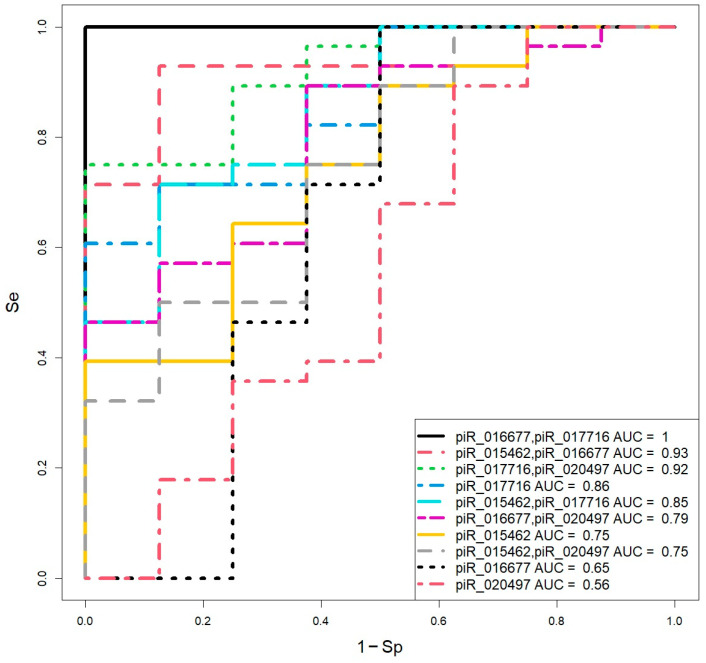
Logistic regression models for assessing day 6 blastocyst quality based on the log2 (fold change) of piwiRNA levels in spent culture media relative to the median “∆∆Ct” in the “EuBl, impl” group.

Logistic regression models for day 6 blastocysts (Figure 4, Table 4) were developed using the same predictor variables. The optimal combinations were piR_016677 and piR_017716 (Se = 100%, Sp = 100%) and piR_015462 and piR_016677 (Se = 93%, Sp = 88%).

The perfect performance (Se = 100%, Sp = 100%, AUC = 1) of the model based on piR_016677 and piR_017716 raised concerns regarding potential overfitting. Due to the limited sample size, an 80%/20% train-test split validation approach was employed [28]; cross-validation was deemed less suitable given the sample size. When the model, retrained on 80% of the data, was applied to the held-out 20% test set, it correctly classified all samples (Table 5) in accordance with Formula (1), supporting the model’s potential utility pending validation on larger, independent cohorts.

(1) $$\frac{1}{1+e^{162-115\;\times \;piR016677\;+\;162\;\times \;piR017716}}$$

### 2.3. Assessment of Blastocyst Quality Irrespective of Developmental Rate

Analysis of piwiRNA levels in blastocysts with different developmental kinetics revealed that euploid blastocysts with implantation potential (EuBl, impl) did not differ significantly (*p* > 0.05) in their piwiRNA profiles based on whether they formed on day 5 or day 6 (Table 6). Specifically, compared to control medium, the spent media from these embryos showed no change in piR_015462 and piR_020497 levels, two-fold increase in piR_016677, and two-fold decrease in piR_017716. Consequently, the median “∆∆Ct” value from the combined cohort of day 5 and day 6 EuBl, impl embryos was established as a universal reference for calculating fold changes in subsequent analyses.

In contrast, poor-quality blastocysts (EuBl, non-impl; AneuBl) exhibited significant differences in piR_020497 and piR_015462 levels depending on the day of blastocyst formation. Specifically, “day 5 EuBl, non-impl” vs. “day 6 EuBl, non-impl” differed in piR_020497 (*p* = 0.022), and “day 5 AneuBl” vs. “day 6 AneuBl” differed in both piR_020497 (*p* < 0.001) and piR_015462 (*p* < 0.001). No significant differences were observed for piR_016677 and piR_017716 levels between day 5 and day 6 poor-quality blastocysts (*p* > 0.05).

Given these findings, a unified model for assessing blastocyst quality (combining ploidy and implantation potential) across both day 5 and day 6 cohorts was developed, using fold change values relative to the combined “EuBl, impl” median.

The most balanced models for the combined dataset were based on the combinations piR_015462 and piR_016677 (Se = 68%, Sp = 71%) and piR_017716 and piR_020497 (Se = 66%, Sp = 71%). The accuracy of these unified models was slightly lower than that of the day-specific models, possibly due to the disparate sample sizes of the combined groups. Therefore, validation of the optimal models from Table 2, Table 4 and Table 7 on an independent test set with a balanced representation of day 5 and day 6 samples is planned.

**Table 7:** Parameters of the logistic regression models presented in Figure 5.

	Wald	*p*_Value	95% CI	OR	Threshold	Se	Sp	Coefficients
(Intercept)	2.801	0.005	0.743 (0.229; 1.276)	2.103 (1.257; 3.581)	0.9338	0.4967	0.9643	0.743
piR_016677	4.381	<0.001	1.115 (0.645; 1.653)	3.049 (1.905; 5.221)				1.115
piR_017716	−3.898	<0.001	−0.938 (−1.451; −0.496)	0.391 (0.234; 0.609)				−0.938
(Intercept)	4.148	<0.001	1.046 (0.562; 1.557)	2.845 (1.754; 4.742)	0.8451	0.6821	0.7143	1.046
piR_015462	−2.815	0.005	−0.569 (−0.981; −0.184)	0.566 (0.375; 0.832)				−0.569
piR_016677	3.523	<0.001	0.758 (0.352; 1.202)	2.135 (1.423; 3.326)				0.758
(Intercept)	4.085	<0.001	1.088 (0.572; 1.625)	2.97 (1.772; 5.079)	0.8505	0.6556	0.7143	1.088
piR_017716	−3.075	0.002	−0.662 (−1.111; −0.255)	0.516 (0.329; 0.775)				−0.662
piR_020497	3.117	0.002	0.913 (0.356; 1.516)	2.491 (1.428; 4.554)				0.913
(Intercept)	8.136	<0.001	1.685 (1.297; 2.112)	5.393 (3.657; 8.263)	0.8524	0.5563	0.8571	1.685
piR_015462	0.553	0.581	0.11 (−0.286; 0.498)	1.116 (0.751; 1.645)				0.11
piR_017716	−1.002	0.316	−0.177 (−0.521; 0.179)	0.837 (0.594; 1.197)				−0.177
(Intercept)	5.86	<0.001	1.368 (0.927; 1.847)	3.928 (2.527; 6.343)	0.7991	0.8609	0.5357	1.368
piR_016677	2.216	0.027	0.365 (0.062; 0.708)	1.44 (1.064; 2.031)				0.365
(Intercept)	5.93	<0.001	1.418 (0.966; 1.908)	4.128 (2.628; 6.74)	0.8311	0.6755	0.5714	1.418
piR_016677	2.443	0.015	0.582 (0.125; 1.065)	1.79 (1.134; 2.902)				0.582
piR_020497	−1.257	0.209	−0.384 (−0.996; 0.209)	0.681 (0.369; 1.232)				−0.384
(Intercept)	8.163	<0.001	1.689 (1.301; 2.116)	5.415 (3.673; 8.294)	0.8576	0.3642	0.9286	1.689
piR_017716	−0.941	0.347	−0.098 (−0.297; 0.119)	0.907 (0.743; 1.126)				−0.098
(Intercept)	6.825	<0.001	1.569 (1.138; 2.043)	4.803 (3.121; 7.717)	0.8294	0.755	0.5714	1.569
piR_020497	0.978	0.328	0.177 (−0.145; 0.566)	1.193 (0.865; 1.761)				0.177
(Intercept)	5.553	<0.001	1.37 (0.904; 1.877)	3.935 (2.468; 6.536)	0.8399	0.6225	0.5357	1.37
piR_015462	−1.778	0.075	−0.368 (−0.79; 0.027)	0.692 (0.454; 1.027)				−0.368
piR_020497	1.994	0.046	0.516 (0.023; 1.044)	1.674 (1.023; 2.84)				0.516
(Intercept)	8.183	<0.001	1.689 (1.302; 2.114)	5.412 (3.675; 8.279)	0.8403	0.7483	0.3571	1.689
piR_015462	−0.411	0.681	−0.05 (−0.28; 0.199)	0.951 (0.756; 1.22)				−0.05

**Figure 5:**
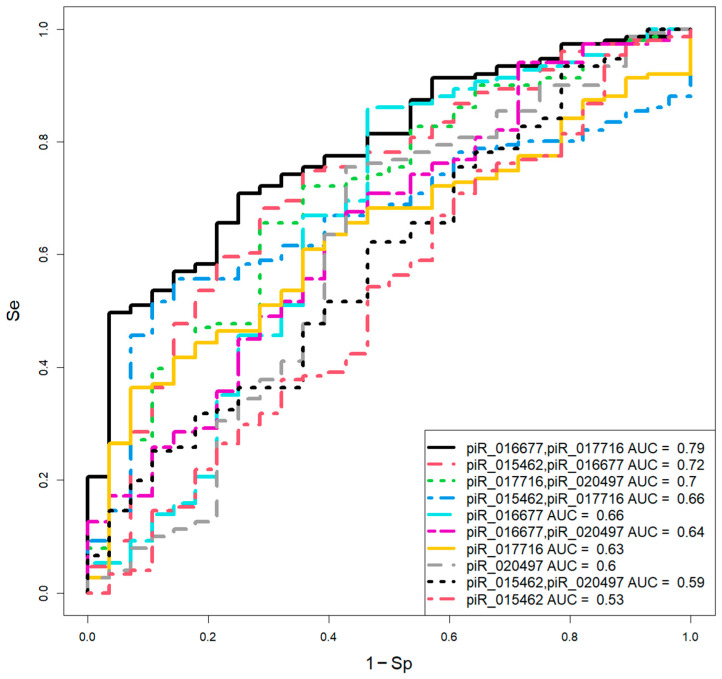
Logistic regression models for assessing blastocyst quality (day 5 and day 6 combined) based on the log2 (fold change) of piwiRNA levels relative to the median “∆∆Ct” in the combined “EuBl, impl” group.

## 3. Discussion

This study successfully validated a set of marker piwiRNA molecules, previously identified by our group [27], on an independent cohort of spent blastocyst culture media. These molecules are associated with critical aspects of blastocyst quality, namely chromosomal ploidy and implantation competence. A key innovation of the present work was the development of logistic regression models tailored to embryos with differing developmental kinetics, specifically those forming late blastocysts on either day 5 or day 6 post-fertilization. Our earlier models for day 5 blastocysts [27] often displayed a marked disparity between sensitivity and specificity, leading to either over-diagnosis of high-potential euploid embryos or over-diagnosis of poor-quality embryos. To address this, we implemented several methodological refinements.

A critical procedural enhancement was the consistent normalization of each spent medium sample against its corresponding lot-matched control medium, incubated under identical temporal and thermal conditions. This step is crucial when using serum-containing media, as different lots can vary substantially in their baseline composition of small non-coding RNAs, including piwiRNAs, and incubation conditions can differentially affect RNA degradation rates, thereby confounding quantitative analysis. The importance of controlling for nucleic acid degradation in spent media is also emphasized in studies utilizing extracellular embryonic DNA for ni-PGT-A [29]. A second key methodological refinement involved the use of a different quantitative metric for inter-group comparisons. We calculated the fold change for each marker piwiRNA in every spent medium sample relative to the median value observed in the reference group of euploid, implantation-competent embryos. This approach biologically contextualizes the data by quantifying the deviation of a given sample from an optimal, validated benchmark. It also helps to mitigate technical variations arising from different threshold determination algorithms inherent to various real-time PCR platforms.

To our knowledge, no other research group has employed piwiRNAs to develop logistic regression models for the simultaneous assessment of embryonic ploidy and implantation potential. We developed specific models for day 5 blastocysts (piR_016677, piR_017716: Se = 69%, Sp = 75%; piR_017716, piR_020497: Se = 73%, Sp = 75%) and for day 6 blastocysts (piR_016677, piR_017716: Se = 100%, Sp = 100%; piR_015462, piR_016677: Se = 93%, Sp = 88%). Furthermore, to streamline the clinical selection of blastocysts for transfer, we identified unique piwiRNA combinations that form the basis of unified models applicable regardless of the day of blastocyst formation (day 5 or 6): piR_015462, piR_016677 (Se = 68%, Sp = 71%) and piR_017716, piR_020497 (Se = 66%, Sp = 71%). The molecules piR_015462 and piR_020497, which showed significant level differences between day 5 and day 6 poor-quality blastocysts, were likely excluded from the top-performing unified models for this reason.

Current non-invasive preimplantation embryo assessment strategies primarily focus on analyzing genomic or mitochondrial DNA from spent culture medium or blastocoelic fluid [29,30,31,32,33,34,35]. While these methods can evaluate ploidy, they provide no direct information on implantation potential. Alternative non-invasive approaches rely on morphological and morphokinetic parameters [36,37,38], which cannot provide a definitive conclusion regarding the ploidy of the embryo and its implantation potential. Notably, embryos with delayed development can still result in successful pregnancies and live births. Reported live birth rates for day 5, 6, and 7 blastocysts are 63%, 51%, and 14%, respectively (Liu et al. [39]), and 69%, 55%, and 36% (Lane et al. [40]). However, the incidence of aneuploidy is higher among day 7 blastocysts (42% euploid) compared to day 6 (54% euploid) and day 5 (63% euploid) blastocysts [40], justifying iPGT-A recommendation for patients with only delayed embryos. The piwiRNA-based RT-PCR and logistic regression models developed herein present a promising alternative for assessing the quality of developmentally delayed blastocysts.

A fundamental function of piwiRNAs is to maintain genomic stability by facilitating the nuclear silencing of retrotransposons within the RISC complex, which recruits histone deacetylases, methyltransferases, and DNA methyltransferases to enact transcriptional repression [41]. In humans, the primary active retrotransposon is *LINE-1*, whose transcription is regulated via CpG methylation and histone deacetylation [42]. Aberrant *LINE-1* expression is linked to genomic instability [43] and has been correlated with meiotic defects and aneuploidy in oocytes [44], as well as defective meiosis and sterility in male germ cells [45]. According to the piRBase database, the marker piwiRNAs identified in this study—piR_016677, piR_017716, piR_020497, and piR_015462—are encoded by *LINE-1* sequences, suggesting their potential role in the epigenetic silencing of *LINE-1* during gametogenesis and early embryogenesis. The mini-review of Mastora et al. [46] emphasizes the relationship between the high concentration of the microinjected unmethylated *LINE-1* DNA and abnormal oocyte morphology, the level of *LINE-1* expression and preimplantation embryo development. Particular attention is paid to the fact that retrotransposition events are necessary for embryogenesis: embryos with repressed *LINE-1* expression show impaired embryonic development, while the induction of retrotransposition events from exogenous retroelements induce DNA double-strand breaks, decreased expression of pluripotency factors (SRY-Box Transcription Factor 2, Sox2; Nanog Homeobox, Nanog), morphological, structural, and cleavage abnormalities in the transfected embryo [46,47]. *LINE-1* is considered as a part of the developmental program of early embryos, the implementation of which requires fine regulation of retrotransposition events, carried out, among others, by piwiRNAs.

Beyond transposon suppression, piwiRNAs regulate cellular signaling pathways through diverse mechanisms [41]. For instance, pachytene piwiRNAs can guide the MIWI protein to target mRNAs, leading to their deadenylation and degradation via deadenylase Caf1 [48], or to direct mRNA cleavage [49]. Using the miRanda algorithm [25], and bioDBnet (https://biodbnet-abcc.ncifcrf.gov/db/db2db.php, accessed on 10 September 2025) for gene symbol conversion, we identified potential mRNA targets for these piwiRNAs (Table S2). Metascape enrichment analysis revealed that the protein products of these target genes are significantly involved in biological processes critical for gametogenesis and early embryogenesis, including male gamete generation, microtubule-based processes, cell morphogenesis, and the Hippo signaling pathway (Table S2). Several target genes exemplify experimentally proven roles in genomic stability, spindle formation, kinetochore function, and cytokinesis—processes whose dysregulation leads to aneuploidy. For example, apolipoprotein B mRNA editing enzyme catalytic subunit 3 (Apobec3), a potential target of hsa_piR_020497, piR_016677, and piR_017716, is involved in defending against uncontrolled *LINE-1* activity during gametogenesis and early development [45]. Polo-like kinase 3 (*Plk3*, a target of hsa_piR_020497) regulates cell cycle progression and cytokinesis [50]. Plk1, a closely related kinase, promotes centrosome disjunction by maintaining serine/threonine-protein kinase (Nek2a) activity, which phosphorylates the centrosomal linker protein rootletin (Crocc, a target of hsa_piR_015462). Mutations in *CROCC* cause severe chromosomal instability and segregation errors [51]. Furthermore, subunits of the Pp2a phosphatase (e.g., Ppp2r5b, a target of hsa_piR_015462) interact with Plk1 and are crucial for maintaining genomic stability [52]; inhibition of *PPP2R5B* perturbs sister chromatid cohesion.

The centrosome serves as a hub for the proteasome and its regulatory components [53]. In eukaryotic cells, the 26S proteasome, comprising a 20S core and a 19S regulatory complexes, degrades the majority of intracellular proteins [54]. Psmd2 (a target of hsa_piR_016677) is a subunit of the 19S complex [55]. Notably, Polo-like kinases themselves are subject to ubiquitin-mediated degradation by the proteasome [50]. This suggests a sophisticated feedback mechanism wherein hsa_piR_016677 and piR_020497 could influence spindle assembly and chromosome segregation by modulating the levels of Psmd2 and Plk3, respectively.

The anaphase-promoting complex/cyclosome (Apc/c), activated by cell division cycle protein 20 homolog (Cdc20) or cadherin 1 (Cdh1, a target of hsa_piR_015462), is another critical cell cycle regulator employing ubiquitin-mediated proteolysis. During oogenesis, Apc/cCdh1 maintains the prophase I arrest of primordial follicles by degrading cyclin B1 [56]. In anaphase I, Apc/cCdh1 facilitates chromosome separation by enabling the phosphorylation and dissociation of the centromeric protein shugoshin 2 (Sgo2) [57]. Deletion of *APC* or *CDH1* in oocytes prevents Sgo2 dissociation and chromosome segregation, leading to aneuploidy.

The piwiRNAs associated with blastocyst aneuploidy in our study also potentially regulate centriolar proteins vital for gametogenesis. Centrosomal Protein 128 (Cep128, target of hsa_piR_015462), centrosomal protein 164 (Cep164, target of hsa_piR_017716), and basomuclin 1 (Bnc1, target of hsa_piR_015462) are key components of the sperm’s maternal centriole appendage, essential for organizing centriolar microtubules and the axoneme [58,59,60,61]. *BNC1* is also critical for oogenesis, as its inhibition allows fertilization but arrests embryonic development at the 2-cell stage [62].

The microtubule-associated protein Map1a (target of hsa_piR_016677) plays a vital role in spermatogenesis by stabilizing microtubule structures within Sertoli cells, which are necessary for the directed transport of developing germ cells [63]. Disruption of this process impairs male fertility. The Ras association domain family member 1 (Rassf1a, target of hsa_piR_015462) promotes microtubule stability by inhibiting histone deacetylase 6 (Hdac6). Stable microtubules are essential for intracellular transport, centrosome/Golgi organization, and focal adhesion stability [64].

During preimplantation development, starting at the 8-cell stage, blastomeres undergo polarization and compaction, processes dependent on cytoskeletal reorganization [65]. The focal adhesion protein paxillin (Pxn, target of hsa_piR_017716) interacts with α-tubulin [66], and its upregulation post-compaction is implicated in forming the adhesive properties necessary for implantation [67]. The adhesion protein Cdh23 (cadherin related 23, target of hsa_piR_016677) regulates microtubule network stability and, together with Plekha7 (pleckstrin homology domain containing A7, target of hsa_piR_015462), stabilizes adhesive intercellular contacts [68,69].

In summary, the selection of abovementioned specific piwiRNAs for quantifying blastocyst quality is strongly supported by their potential biological roles, spanning from genomic stability maintenance via *LINE-1* regulation to direct involvement in cell cycle control, spindle assembly, and the cellular adhesion processes imperative for successful implantation.

## 4. Materials and Methods

### 4.1. Patient Cohort

The study enrolled couples undergoing IVF/ICSI with PGT-A cycles at the National Medical Research Center for Obstetrics, Gynecology and Perinatology named after Academician V.I. Kulakov in 2025. Standard GnRH-antagonist or GnRH-agonist protocols were used. Inclusion criteria were: infertility duration ≥ 1 year, and indications for PGT-A including advanced maternal age (≥35 years), recurrent pregnancy loss (≥2 losses), previous unsuccessful ART cycles, or severe male factor (sperm concentration < 5 million/mL, and/or progressive motility (a + b) < 19%, and/or normal morphology < 1%). All contraindications for ART and pregnancy per Russian Ministry of Health Order No. 803n (31 July 2020) served as exclusion criteria. Written informed consent was obtained from all participants. The study was approved by the local Ethics Committee (Protocol No. 1, 30 January 2025).

### 4.2. Collection of Spent Embryo Culture Medium Samples

Embryos were cultured in COOK (Australia) multigas incubators. Blastocyst quality was assessed on days 5 and/or 6 using standard morphological criteria [68]. In parallel, the spent culture media from embryos undergoing PGT-A were collected on day 5 or 6 for embryos reaching the late blastocyst stage, and excellent/good quality blastocysts underwent trophectoderm biopsy followed by vitrification. Biopsied cells were lysed for analysis. PGT-A was performed using next-generation sequencing (NGS). Euploid embryos were transferred into the uterine cavity in subsequent frozen cycles following endometrial preparation. Serum β-hCG was measured 10 days post-transfer, and clinical pregnancy was confirmed by ultrasound 21 days post-transfer. Embryo quality, PGT-A results, and ART outcomes are detailed in Table S1.

### 4.3. RNA Isolation from Culture Medium Samples

RNA was isolated from 5 to 25 μL of spent or control culture medium (adjusted to 200 μL with 0.9% NaCl) using the miRNeasy Serum/Plasma Kit (Qiagen, Hilden, Germany) according to the manufacturer’s instructions.

### 4.4. Reverse Transcription

Two microliters of total RNA eluate were reverse transcribed using the miRCURY LNA RT Kit (Qiagen, Hilden, Germany) for Probe PCR. cDNA was diluted 1:40 and stored at −80 °C.

### 4.5. Quantitative Real-Time PCR

qPCR was performed using 2 μL of cDNA, miRCURY Probe PCR reagents (Qiagen), 1x Universal antisense primer (Qiagen), 1X qPCRmix-HS SYBR + HighROX (Evrogen, Moscow, Russia), and 0.2 µM of a target-specific sense primer (Evrogen; sequences in Table 8) on a StepOnePlus™ thermocycler (Applied Biosystems, Waltham, MA, USA). Cycling conditions were: 95 °C for 15 min; 40 cycles of 94 °C for 15 s, 52.7 °C for 30 s, 70 °C for 30 s. Melt curve analysis confirmed specificity. Relative piwiRNA expression was calculated by the ∆∆Ct method using hsa_piR_022258 as the reference and a no-embryo control medium as the calibrator.

### 4.6. Statistical Analysis

Data analysis was performed using custom scripts in R [70] within RStudio [71]. Normality was assessed using the Shapiro–Wilk test. Non-normally distributed data were compared using the Mann–Whitney U-test and described as median (Me) and quartiles (Q1; Q3). A *p*-value < 0.05 was considered significant; *p* < 0.001 is indicated as such.

Logistic regression was used to build predictive models. Model performance was evaluated via ROC analysis, determining the area under the curve (AUC), sensitivity, specificity, and an optimal cut-off point. Stepwise feature selection in RStudio was used to identify the most contributory piwiRNA predictors for embryo quality.

## 5. Conclusions

A non-invasive method for identifying euploid blastocysts with high implantation potential has been developed for the effective implementation of assisted reproductive technology programs on the day of late blastocyst formation, namely on the 5th or 6th day after fertilization, based on the content of hsa_piR_016677, hsa_piR_017716, hsa_piR_020497, hsa_piR_015462, and hsa_piR_022258 in the spent culture medium using quantitative RT-PCR (polymerase chain reaction coupled with reverse transcription) in real time. It can be used by a reproductive physician or embryologist to select the highest quality embryo for transfer into the uterine cavity in a superovulation stimulation cycle without the need forced cryopreservation of embryos, since it will ensure obtaining the analysis result and transfer of the selected embryo on the same day. If there are indications for a married couple to undergo preimplantation genetic testing for aneuploidies (PGT-A), it can be used to screen all blastocysts obtained in the superovulation stimulation cycle in order to select the highest quality embryo(s) for PGT-A, their cryopreservation and further transfer in a natural cycle or with the use of cyclic hormone therapy.

## Figures and Tables

**Table 1:** Log2(fold change) in piwiRNA levels in spent culture media from day 5 blastocysts relative to the median “∆∆Ct” value in the “EuBl, impl” group.

piwiRNA	Group	Me (Q1;Q3) *	EuBl, impl **	EuBl, Non-impl **	AneuBl **
piR_016677	EuBl, impl	0 (−0.54; 1.17)	1	0.04	0.02
	EuBl, non-impl	0.99 (0.1; 2.65)	0.04	1	0.9
	AneuBl	1.01 (0.36; 1.9)	0.02	0.9	1
piR_017716	EuBl, impl	0 (−0.74; 0.58)	1	0.78	0.16
	EuBl, non-impl	−0.34 (−0.98; 1.98)	0.78	1	0.15
	AneuBl	−0.62 (−1.35; 0.75)	0.16	0.15	1
piR_020497	EuBl, impl	0 (−0.29; 0.95)	1	0.02	0.1
	EuBl, non-impl.	1.02 (0.37; 1.57)	0.02	1	0.04
	AneuBl	0.6 (0.15; 1.1)	0.1	0.04	1
piR_015462	EuBl, impl	0 (−1.04; 0.9)	1	0.9	0.5
	EuBl, non-impl	0.16 (−0.91; 0.74)	0.9	1	0.48
	AneuBl	0.27 (−0.76; 1.03)	0.5	0.48	1

* Values are presented as Me (Q1; Q3), where Me is the median, Q1 is the 25th percentile, and Q3 is the 75th percentile. ** Statistical significance of differences was assessed using the Mann–Whitney U-test.

**Table 2:** Parameters of the logistic regression models presented in Figure 2.

	Wald	*p*-Value	95%CI	OR	Threshold	Se	Sp	Coefficients
(Intercept)	3.594	<0.001	1.055 (0.494; 1.655)	2.873 (1.639; 5.232)	0.84	0.69	0.75	1.055
piR_016677	3.364	<0.001	0.967 (0.441; 1.586)	2.63 (1.554; 4.883)				0.967
piR_017716	−2.743	0.006	−0.769 (−1.376; −0.259)	0.464 (0.253; 0.772)				−0.769
(Intercept)	3.658	<0.001	1.157 (0.546; 1.801)	3.179 (1.727; 6.053)	0.85	0.73	0.75	1.157
piR_017716	−2.43	0.015	−0.578 (−1.074; −0.121)	0.561 (0.341; 0.886)				−0.578
piR_020497	2.782	0.005	0.955 (0.302; 1.667)	2.598 (1.353; 5.298)				0.955
(Intercept)	5.02	<0.001	1.388 (0.868; 1.962)	4.009 (2.383; 7.112)	0.85	0.68	0.71	1.388
piR_015462	−1.211	0.226	−0.269 (−0.719; 0.16)	0.764 (0.487; 1.174)				−0.269
piR_016677	2.344	0.019	0.542 (0.103; 1.017)	1.719 (1.109; 2.764)				0.542
(Intercept)	5.577	<0.001	1.494 (0.992; 2.05)	4.453 (2.697; 7.767)	0.82	0.84	0.55	1.494
piR_016677	2.027	0.043	0.364 (0.037; 0.742)	1.439 (1.037; 2.101)				0.364
(Intercept)	5.414	<0.001	1.526 (0.999; 2.111)	4.599 (2.717; 8.261)	0.84	0.71	0.61	1.526
piR_016677	1.638	0.101	0.442 (−0.073; 0.993)	1.557 (0.929; 2.698)				0.443
piR_020497	−0.39	0.697	−0.144 (−0.878; 0.576)	0.866 (0.416; 1.778)				−0.144
(Intercept)	5.752	<0.001	1.589 (1.074; 2.164)	4.899 (2.928; 8.709)	0.83	0.79	0.61	1.589
piR_020497	1.35	0.177	0.308 (−0.089; 0.805)	1.36 (0.915; 2.237)				0.308
(Intercept)	7.314	<0.001	1.782 (1.329; 2.289)	5.94 (3.776; 9.866)	0.87	0.53	0.75	1.782
piR_015462	1.249	0.212	0.281 (−0.164; 0.725)	1.324 (0.849; 2.066)				0.281
piR_017716	−1.1	0.271	−0.222 (−0.617; 0.184)	0.801 (0.539; 1.202)				−0.222
(Intercept)	5.238	<0.001	1.523 (0.981; 2.131)	4.584 (2.668; 8.423)	0.81	0.91	0.32	1.523
piR_015462	−0.639	0.523	−0.145 (−0.604; 0.291)	0.865 (0.547; 1.338)				−0.145
piR_020497	1.444	0.149	0.438 (−0.137; 1.061)	1.549 (0.872; 2.888)				0.438
(Intercept)	7.515	<0.001	1.811 (1.37; 2.325)	6.17 (3.935; 10.223)	0.86	0.38	0.85	1.82
piR_017716	−0.179	0.858	−0.022 (−0.250; 0.241)	0.978 (0.778; 1.273)				−0.022
(Intercept)	7.406	<0.001	1.795 (1.344; 2.299)	6.019 (3.835; 9.969)	0.85	0.63	0.51	1.795
piR_015462	0.618	0.537	0.091 (−0.182; 0.399)	1.095 (0.833; 1.490)				0.091

**Table 3:** Log2(fold change) in piwiRNA levels in spent culture media from day 6 blastocysts relative to the median “∆∆Ct” value in the “EuBl, impl” group.

piwiRNA	Group	Me (Q1; Q3) *	EuBl, impl **	EuBl, Non-impl **	AneuBl **
piR_016677	EuBl, impl	0 (−0.61; 1.16)	1	0.5	0.2
	EuBl, non-impl	0.58 (0.44; 1.06)	0.5	1	0.67
	AneuBl	0.78 (0.47; 1.16)	0.2	0.67	1
piR_017716	EuBl, impl	0 (−0.57; 1.87)	1	0.01	0.003
	EuBl, non-impl	−0.82 (−0.91; −0.74)	0.01	1	0.97
	AneuBl	−0.88 (−1.4; −0.45)	0.003	0.97	1
piR_020497	EuBl, impl	0 (−0.27; 1.21)	1	0.85	0.4
	EuBl, non-impl	0.13 (0.06; 0.31)	0.85	1	0.14
	AneuBl	−0.1 (−0.48; 0.34)	0.4	0.14	1
piR_015462	EuBl, impl	0 (−0.73; 1.15)	1	0.57	0.015
	EuBl, non-impl	−0.34 (−0.8; 0.22)	0.57	1	0.1
	AneuBl	−0.84 (−1.66; −0.44)	0.015	0.1	1

* Values are presented as Me (Q1; Q3).; ** Statistical significance of differences was assessed using the Mann–Whitney U-test.

**Table 4:** Parameters of the logistic regression models presented in Figure 4.

	Wald	*p*_Value	95%CI	OR	Threshold	Se	Sp	Coefficients
(Intercept)	−0.001	0.999	−179.213 (−82,930.486; 20,089.428)	1.47 × 10^−78^ (0; Inf)	0.5	1	1	−179.213
piR_016677	0.001	0.999	131.019 (−4729.481; 4991.519)	7.96 × 10^56^ (0; Inf)				131.019
piR_017716	−0.002	0.999	−177.891 (−73,828.946; −162,647.348)	5.53 × 10^−78^ (0; 0)				−177.891
(Intercept)	−0.973	0.331	−0.891 (−3.013; 0.701)	0.41 (0.049; 2.034)	0.75	0.93	0.88	−0.891
piR_015462	−2.945	0.003	−2.214 (−4.068; −1.008)	0.109 (0.017; 0.365)				−2.214
piR_016677	2.467	0.014	2.537 (0.872; 5.031)	12.646 (2.392; 153.113)				2.537
(Intercept)	−0.877	0.381	−1.072 (−4.125; 0.898)	0.342 (0.016; 2.455)	0.83	0.75	1	−1.072
piR_017716	−2.027	0.043	−5.369 (−12.553; −1.741)	0.005 (3.54 × 10^−6^; 0.175)				−5.369
piR_020497	1.909	0.051	2.088 (0.193; 4.735)	8.061 (1.213; 113.859)				2.088
(Intercept)	0.179	0.858	0.139 (−1.554; 1.505)	1.149 (0.211; 4.505)	0.89	0.61	1	0.139
piR_017716	−2.058	0.04	−2.476 (−5.52; −0.847)	0.084 (0.004; 0.429)				−2.476
(Intercept)	0.135	0.893	0.107 (−1.609; 1.499)	1.113 (0.201; 4.479)	0.86	0.71	0.88	0.107
piR_015462	0.431	0.667	0.407 (−1.36; 2.496)	1.502 (0.257; 12.138)				0.407
piR_017716	−1.758	0.079	−2.911 (−7.128; −0.595)	0.054 (0.001; 0.551)				−2.911
(Intercept)	1.247	0.212	0.683 (−0.412; 1.797)	1.981 (0.662; 6.034)	0.7	0.89	0.62	0.683
piR_016677	1.991	0.047	1.438 (0.159; 3.109)	4.213 (1.172; 22.402)				1.438
piR_020497	−2.184	0.029	−1.606 (−3.289; −0.307)	0.201 (0.037; 0.736)				−1.606
(Intercept)	2.044	0.041	0.962 (0.046; 1.937)	2.618 (1.047; 6.936)	0.67	0.89	0.5	0.962
piR_015462	−2.112	0.035	−0.992 (−2.174; −0.25)	0.371 (0.114; 0.779)				−0.992
(Intercept)	1.665	0.096	0.811 (−0.145; 1.815)	2.251 (0.865; 6.139)	0.68	0.89	0.5	0.811
piR_015462	−2.227	0.026	−1.394 (−2.902; −0.321)	0.248 (0.055; 0.726)				−1.394
piR_020497	1.009	0.313	0.735 (−0.633; 2.436)	2.086 (0.531; 11.43)				0.735
(Intercept)	1.991	0.046	0.987 (0.056; 2.048)	2.684 (1.058; 7.753)	0.7	1	0.5	0.987
piR_016677	0.815	0.415	0.413 (−0.529; 1.498)	1.512 (0.589; 4.471)				0.413
(Intercept)	3.179	0.002	1.377 (0.593; 2.323)	3.965 (1.809; 10.209)	0.9	1	0.13	1.378
piR_020497	−1.329	0.184	−0.591 (−1.573; 0.271)	0.553 (0.207; 1.311)				−0.591

**Table 5:** Performance of the model (based on Formula 1, retrained on 80% of data) on the 20% test set of spent culture media from day 6 blastocysts.

Blastocyst Quality	piR_016677	piR_017716	Model Result (Threshold = 0.5)
EuBl, impl	2.313089	2.359285	6 × 10^−122^
EuBl, impl	−0.5407	0.318697	1 × 10^−120^
EuBl, non-impl	0.320295	−0.89333	1
AneuBl	−0.04268	−2.0471	1
AneuBl	1.12153	−0.5075	1
AneuBl	−0.26189	−1.36328	1
AneuBl	1.100154	−0.31455	1
AneuBl	2.194816	−0.11771	1

**Table 6:** Median fold change values for piwiRNA levels in spent culture media from euploid blastocysts with high implantation potential on day 5, day 6, and combined.

	piR_015462	piR_016677	piR_017716	piR_020497
		5 day EuBl, impl		
∆∆Ct	−0.030	1.186	−1.194	0.434
Fold Change *	0.979	2.276	0.437	1.351
		6 day EuBl, impl		
∆∆Ct	−0.488	1.068	−0.882	0.294
Fold Change *	0.713	2.097	0.542	1.226
		5 + 6 day EuBl, impl		
∆∆Ct	−0.258	1.186	−1.180	0.403
Fold Change *	0.836	2.276	0.441	1.322

* Fold change was calculated as 2^(∆∆Ct).^

**Table 8:** Nucleotide sequences of sense primers used for piwiRNA quantification by qRT-PCR.

piwiPHK ^1^	Identification Number ^1^	Nucleotide Sequence of the Sense Primer for PCR, 5′-3′
hsa_piR_015462	DQ591122	tgtcctgggccagcctgatgatgtcctcctc
hsa_piR_016677	DQ592953	cccctggtggtctagtggttaggattcggc
hsa_piR_020497	DQ598177	ggggggtgtagctcagtggtagagcgcgtgct
hsa_piR_017716	DQ594453	ttccctggtggtctagtggttaggattcggc
hsa_piR_022258	DQ600471	tactacctgattggtcgggtgtgagc

^1^ piRBase http://bigdata.ibp.ac.cn/piRBase/name_convert.php, accessed on 10 September 2025.

## Data Availability

The data presented in this study are available in this article and Supplementary Materials.

## Supplementary Materials

The following supporting information can be downloaded at: https://www.mdpi.com/article/10.3390/ijms262412102/s1.
